# Long-Term Cell-Membrane-Coated Ultrabright Nanospheres for Targeted Cancer Cell Imaging and Hydrophobic Drug Delivery

**DOI:** 10.1021/acs.chemmater.4c01819

**Published:** 2025-01-30

**Authors:** Rajendra Prasad, Berney Peng, Narendra Gupta, Avtar Singh Meena, Geetha Satya Sainaga Jyothi Vaskuri, Anuj Chandak, Igor Sokolov, João Conde

**Affiliations:** a School of Biochemical Engineering, Indian Institute of Technology (BHU), Varanasi, Uttar Pradesh 221005, India; b Departments of Mechanical Engineering and Biomedical Engineering, 1810 Tufts University, Medford, Massachusetts 02155, United States; c Vasco Healthcare Pvt., Ltd., Gopal Pura Bai Pass, Jaipur, Rajasthan 303006, India; d Department of Biotechnology, 28730 All India Institute of Medical Sciences, Ansari Nagar, New Delhi 110029, India; e Pharmaceutical Sciences Department, College of Pharmacy, University of Tennessee Health Science Center, Memphis, Tennessee 38163, United States; f NOVA Medical School|Faculdade de Ciências Médicas, NMS|FCM, 56070 Universidade NOVA de Lisboa, 1169-056 Lisbon, Portugal; g Comprehensive Health Research Centre, NOVA Medical School|Faculdade de Ciências Médicas, NMS|FCM, 56070 Universidade NOVA de Lisboa, 1169-056 Lisbon, Portugal

## Abstract

Nanoparticle-based imaging agents have gained massive attention for the targeted cancer cell imaging of early stage disease diagnosis. Among these, organic dye-entrapped and -assembled nanoparticles have been considered as potential imaging agents. However, they are limited by poor brightness, low stability, low reproducibility, and selective surface engineering, which limit their translational potential. The molecular assembly of amphiphilic precursors and the chosen organic fluorophore can augment the brightness and stability of the engineered nanoimaging agents. Herein, we describe cancer cell-membrane-covered ICG-cellulose acetate nanospheres (180 nm) as biomimetic ultrabright nanoimaging agents for cancer cell imaging. Engineered biomimetic ultrabright imaging agents are compared with folic-acid-conjugated ultrabright nanospheres. Encapsulation of fluorescent organic molecules (660 dye molecules/per nanoparticle) in the core of a polymeric network enhances the overall brightness and long-term photostability due to the unique assembly of the loaded fluorescent cargo and poor permeation of oxygen to oxidize the dye. The amphiphilic nature of the selected polymeric network accommodates both hydrophilic and hydrophobic cargo molecules (e.g., imaging and therapeutics). The engineered fluorescent nanoparticles exhibit high brightness, uniform particle size distribution, high stability, good biocompatibility with normal cells, and high scalability. For targeted chemotherapeutics, DOX-loaded biomimetic nanospheres demonstrate better chemotherapeutic response (more than 95% cancer cell death) than folic acid-attached DOX-loaded nanoparticles (78% cancer cell death). The engineered nanospheres exhibited cancer cell imaging and therapeutics capabilities by delivering imaging and drug molecules in cancer-mimicked environment *in vitro*. Our findings suggest that the engineered ultrabright nanospheres not only overcome the limitations of nanoimaging but also provide additional advantages for targeted cancer therapeutics.

## Graphical abstract

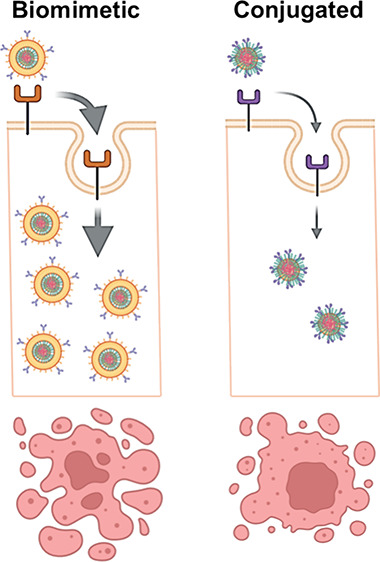

## Introduction

Nanoparticles have gained extensive attention as imaging agents^1–3^ for various imaging modalities such as fluorescence imaging, contrast imaging, magnetic resonance imaging, photoacoustic imaging, ultrasound imaging, etc.^4–7^ Moreover, nanoparticles (metallic and nonmetallic) have been recognized as versatile platforms for detecting cancer at early stage,^8,9^ image-guided surgery,^10–13^ and many of them have been used as cargo carriers.^14–16^ It should be noted that some nanosized imaging agents have been tested clinically and considered by the US FDA.^17–24^ Among them, optically active emissive/fluorescent nanoparticles have been considered as potent imaging agents due to small size, confined electronic properties, unique optical properties, etc.^25–27^ However, making them ultrabright and optically stable for a long time are yet to be achieved.^25,26^ On the other hand, nanoparticles based on both imaging and therapeutic systems still suffer from biocompatibility, degradation, low colloidal stability, uncontrolled particle size distribution, poor product yield, and reproducibility, etc., though nanoparticles have been considered for clinical trials.^21,22^ Further, pristine nanoparticles^28–34^ rarely show fluorescent properties except for quantum dots (QDs),^35–40^ but QDs are less recommended for biomedical applications due to toxicity and nonspecific biodistribution.^37,40^ Fluorescent organic molecules have been considered promising imaging probes, but they face major limitations in terms of easy degradation, low optical stability and poor circulation.^27,41,42^ Therefore, nanoparticle integration has been propsoed to improve their (i) optical stability, (ii) maintain emission property for a long time, (iii) improve blood circulation time (hours to days), (iv) localized targeting ability of desire site, (v) specific biodistribution upon *in vivo* administration, etc.^43–45^ It is well documented in the literature that fluorescent molecules within nanoparticles entity are integrated by just encapsulation/entrapment, chemical conjugation, and physical interaction.^46–49^ So far, fluorescent dye-encapsulated nanoparticles such as mesoporous silica,^28,47,48^ polymeric nanospheres,^46,49–54^ liposomes,^55^ gold nanoparticles,^56^ metal–organic frameworks,^57^ etc. have been designed and tested for bioimaging applications. However, two major parameters of these nanoparticles, viz., (i) ultra brightness^28–34^ and (ii) integrating imaging and chemotherapeutic drugs within a single nanoparticle, are less explored.^44^ Achieving ultrabrightness^28–34^ is always a significant concern in the case of nanoparticle-based imaging agents, which depends on the unique assembly of fluorescent molecules within the nanoparticle network, as described below in detail. Second, entrapping chemotherapeutic drug molecules requires an extra space or entity within the nanoparticle, which can be achieved by using an amphiphilic framework. For example, liposomal nanoparticles can accommodate both hydrophilic and hydrophobic imaging and therapeutic cargo molecules,^58–60^ but they suffer from low stability due to the fragile nature of lipid^58^ self-assembly. Therefore, surface engineering approaches^60^ have been attempted to overcome the limitations of liposomal nanoparticles,^58^ but they are complicated and time-consuming. However, we are still limited to unanswered questions such as: (1) Can we design a simple nanoparticle that can accommodate both imaging and therapeutic molecules? (2) Can we achieve ultrabright nanoimaging agents that maintain their optical stability for a long time? (3) Can we evaluate the image-guided chemotherapeutic response in cancer cells?

To address the major issues associated with an ideal nanoimaging agent, we chose a clinically safe polymeric network assembly, cellulose acetate (CA)^26,61^ and polyethylene glycol (PEG). The unique assembly of CA and PEG produces nanospheres (CA-dots) with amphiphilic properties that can accommodate both hydrophilic and hydrophobic small molecules (imaging and therapeutic agents, respectively). The specific orientation of fluorescent organic dye molecules within the deep hydrophobic core of nanoparticle network induces ultrabrightness, whereas the outer hydrophilic layer conjugates the targeting ligands via coupling chemistry.^26,61^ Moreover, the amphiphilic chemistry of engineered polymeric nanoparticles allows cancer cells to be covered with cell membrane NanoGhosts to produce biomimetic nanoparticles. Such a system offers site-selectivity and natural targeting ability for targeted cancer cell imaging and therapy.

## Results and Discussion

### Engineering Biomimetic and Conjugated Nanospheres

Cancer cell (colon cancer cell) membrane NanoGhosts, named NGs, were used to prepare the biomimetic ultrabright nanospheres, as shown in the Supporting Information Figure S1. First, ultrabright nanospheres also named as nanodots (N-dots) were engineered by assembling fluorescent organic dye ICG (0.01 mM) within the core of a supramolecular cellulose acetate (CA)-supported polymer guest (Pluronic F127, PF127) via nanoprecipitation, as shown in Figure 1A. The dialyzed nanoparticles were characterized by a copious number of physicochemical techniques, showing spherical morphology with a diameter of 180 nm (Figure 1B,C). During nanoprecipitation, the incorporated guest polymeric PF127 chains controlled the morphology and size of the nanoparticles, which was corroborated by dynamic light scattering measurements (0.22 PDI), as shown in Figure 1D. Moreover, the hydrophilic outer layer of PF127 improved the colloidal stability of the prepared nanoparticles, whereas the diffused hydrophobic moieties of the integrated block copolymer strengthened the nucleating CA core for organic dye entrapment, as validated through optical characterization. A negative surface charge (zeta potential of −12 mV) indicates better stability of the designed nanoparticles, which exhibit better dispersion with improved negative surface charge after folic acid conjugation (−18 mV) and cell-membrane ghost nanovesicle covering (−22 mV), as shown in Figure 1E. The photonic and photostability properties of ICG-encapsulated biomimetic nanodots (ICG@nanospheres-NGs) were examined by optical characterization, where specific encapsulation of ICG dye molecules maintained the ultra brightness and photostability of the engineered nanoparticles. From the absorption spectra, we noted that free ICG dye molecules exhibited the maximum absorbance at 701 nm, whereas peak broadening with a red shift was observed in the case of ICG-encapsulated biomimetic nanospheres (782 nm) and NGs (794 nm) nanoparticles (Figure 1F,G). Free ICG showed absorbance around 780 nm at low concentration, but in the present study, we noted low absorbance with blue shift (701 nm) due to used high concentration of ICG in aqueous, i.e., 0.01 mM, which also caused aggregation. However, ICG-entrapped polymeric nanoparticles resulted red shift (782 nm) with promising scattering may be due to homogeneous encapsulation of ICG molecules within polymeric network “nanospheres”. In addition to characteristic absorption peak in near infrared range, a specific absorption peak at 585 nm indicated ICG encapsulation within NGs, which was not observed in the case of free ICG molecules (Figure 1G). After being purified via dialysis (for 2 days using a 12 KD dialysis membrane), the engineered nanoparticles showed promising photoluminance properties with a bathochromic shift in emission (λ_em_ 824 nm) compared to free ICG dye molecules (λ_em_ 820 nm) upon their integration with biomimetic cell ghost nanovesicles (Figure 1H). Time-dependent (1–800 h) optical properties were measured to evaluate the photostability of ICG dye-entrapped nanoparticles, as shown in Figure 1I. ICG dye-entrapped nanoparticles (nanospheres and biomimetic nanospheres) maintained their optical response even after 33 days of storage due to the tight entrapment of loaded fluorescent dye molecules in the deep core of the nanoparticles. However, the optical stability of free dye molecules was hampered by long-term storage, owing to light sensitivity and degradation.

**Figure 1:**
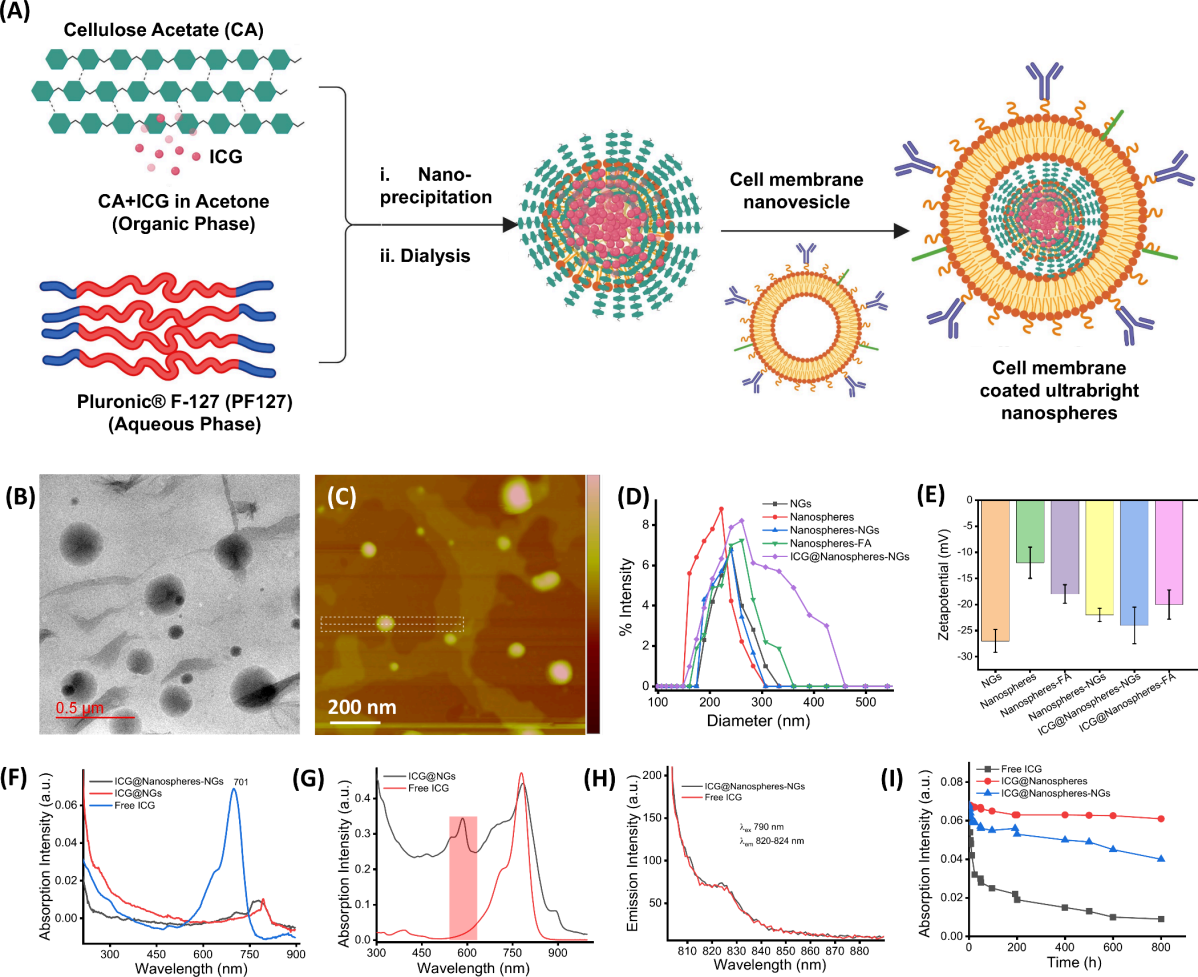
(A) Schematic representation of biomimetic ultrabright nanoparticles followed by nanoprecipitation, (B, C) TEM and AFM images of biomimetic ultrabright nanoparticles, (D, E) dynamic light scattering measurements and surface charge measurements of biomimetic ultrabright nanoparticles and related nanoparticulate formations (NGs, nanospheres, nanospheres-NGs, nanospheres-FA, etc.), (F–H) UV–vis–NIR absorption and emission spectra of engineered biomimetic ultrabright nanoparticles and free dye molecules, and (I) time-dependent photostability measurements of engineered ultrabright polymeric nanoparticles and biomimetic ultrabright nanoparticles.

To assemble targeted ligand-conjugated ultrabright nanospheres, folic acid was coupled to PEG chains of the guest polymer PF127, followed by coupling chemistry under ambient conditions. Nanoprecipitation of the organic phase (CA mixed with ICG in an organic solvent) with the aqueous phase of the conjugated guest polymer PF127 resulted in nanospheres, as shown in Figure 2A. The prepared nanoparticles were purified via dialysis against an aqueous medium for 2 days and validated using microscopic and spectroscopic characterizations. The obtained nanoparticles showed spherical morphology (220–240 nm) as seen from transmission electron microscopy and atomic force microscopic imaging, which was further supported by the dynamic light scattering (DLS) measurements (0.38 PDI), as shown in Figure 2B–D. A significantly higher negative surface charge (−20 mV) indicated folic acid conjugation compared to free nanospheres (−12 mV), which was further increased (−22 mV) after the fluorescent organic dye ICG was encapsulated (Figure 2E). Moreover, the high negative surface charge (−22 to −24 mV) ensured the stability of the conjugated nanoparticles even after long storage (30 days). Similarly, peak broadening and red shift in the optical spectra indicated the encapsulation of the fluorescent ICG dye in the deep core of the conjugated nanoparticles, as shown in Figure 2E,F. In the absorption spectra, the dye-encapsulated nanoparticles showed about a 20 nm red shift (770–790 nm) compared to free ICG dye. A specific sharp peak around 900 nm indicated the aggregated form of ICG dye molecules, which was maintained with broadness after encapsulating these aggregated ICG molecules within the folic acid-conjugated nanospheres (nanospheres-FA). Multiple peaks between 690 and 900 nm in the absorption spectra indicated the aggregation of dye molecules, which may be due to the long storage time. The absorption peak around 367 nm presented π–π* electron transitions, which were due to the conjugation of folic acid molecules with the nanospheres. Moreover, the folic acid conjugation was verified through FTIR spectroscopic measurements. It showed stretching and vibrational bands of N–H bonds between 610 and 830 cm^–1^ and 1585–3340 cm^–1^. Peaks at 1690 and 1640 cm^–1^ represent the −C=O and −C=N groups of the pterin structure in folic acid, respectively. 1595 and 1478 cm^–1^ peaks were due to the −CONH_2_ bending and −C=C stretching of the phenyl and pterin rings in folic acid, respectively, as shown in the Supporting Information, Figure S2. These conjugated nanoparticles exhibited near-infrared emission (λ_em_ 812 nm) (Figure 2G). Strong encapsulation of fluorescent ICG dye molecules in the deep core of conjugated nanoparticles (nanospheres-FA) demonstrated significantly higher photostability than free ICG dye molecules, as shown by the time-dependent (1–800 h) optical properties (Figure 2H). We understood that the prepared fluorescent conjugated nanoparticles showed promising brightness (678–702 MESF unit), see Figure 2I. To the best of our knowledge, the reason behind the higher brightness or “ultrabrightness” of dye-encapsulated nanoparticles (ICG@nanospheres-NGs and ICG@nanospheres-FA) was their polymeric network, which protects the encapsulated dye from dimerization and fluorescence quenching.^62^

**Figure 2:**
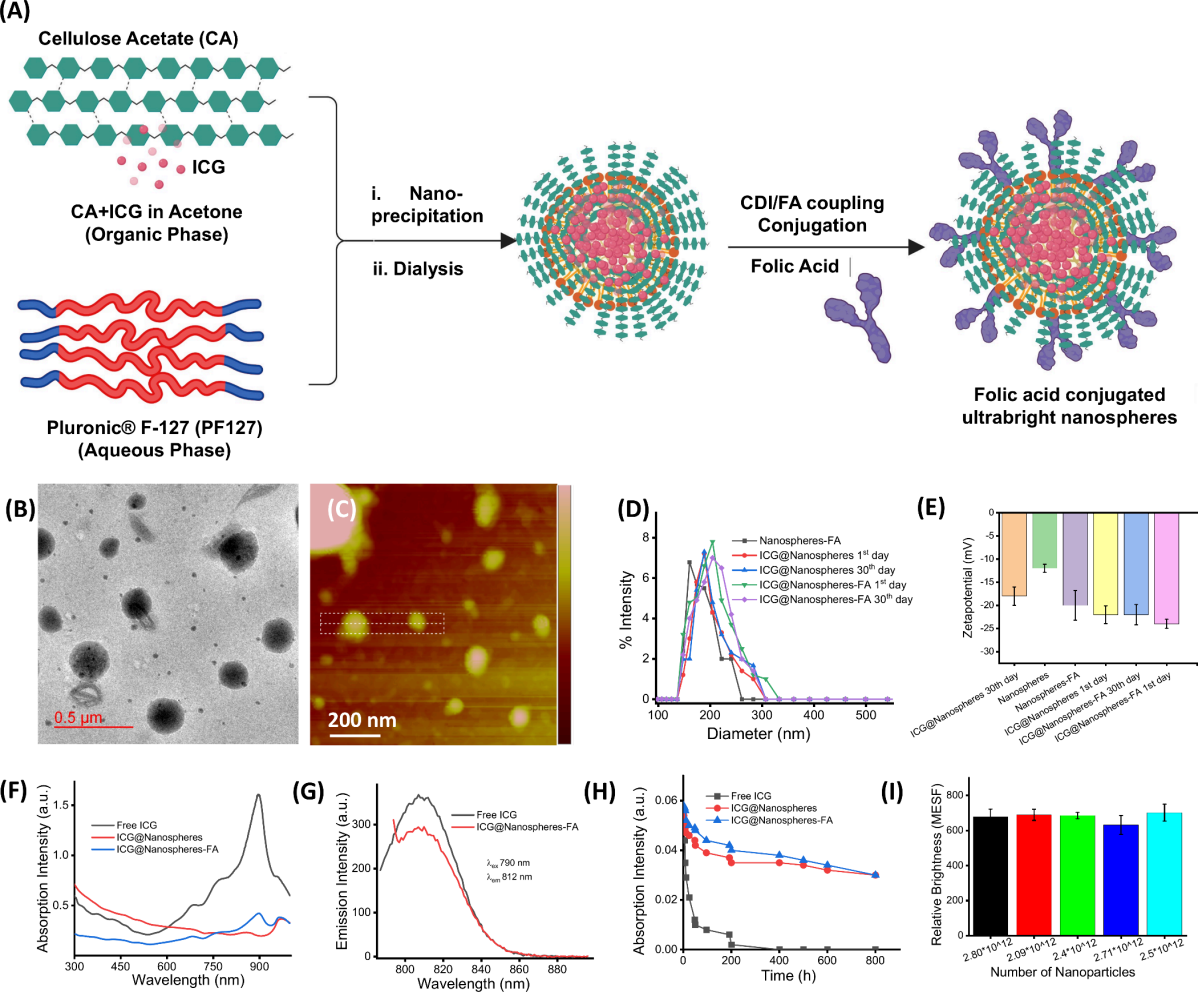
(A) Schematic representation of folic acid-conjugated ultrabright nanoparticles followed by nanoprecipitation, (B, C) TEM and AFM images of folic acid-conjugated ultrabright nanoparticles, (D, E) dynamic light scattering measurements and surface charge measurements of folic acid-conjugated ultrabright nanoparticles and related nanoparticulate formations, (F, G) UV–vis–NIR absorption and emission spectra of engineered folic acid-conjugated ultrabright nanoparticles and free dye molecules, (H) time-dependent photostability measurements of folic acid-conjugated ultrabright nanoparticles, and (I) relative brightness of folic acid-conjugated ultrabright nanoparticles at various concentrations (in terms of number of nanoparticles per cm^3^).

### Characteristics of Biomimetic Ultrabright Nanodots

To the best of our understanding, the specific orientation and distance between dye molecules in the core of polymeric network altered the ultrabrightness of engineered nanoparticles. The number of encapsulated dye molecules per nanoparticle and their relative brightness (MESF, i.e., molecules of equivalent soluble fluorophore) were calculated by drying (50 °C) a known volume (200 μL) of nanoparticles (180–220 nm diameter), as shown in Figure 3. We obtained a 3.9 × 10^–12^ mg weight of one nanoparticle that accommodated approximately 900 ICG dye molecules within a single nanoparticle. Spectroscopic studies were performed to calculate the number of dye molecules per cm^3^ (4.59 × 10^13^ dye molecules/cm^3^) considering the Beer–Lambert law. For relative brightness, fluorescence was measured from the ICG-encapsulated nanoparticles (biomimetic and conjugated nanospheres) and the same concentration of the reference dye ICG. The calculated brightness was in MESF units (molecules of equivalent soluble fluorophores), which are well-stated units used in flow cytometry. Both biomimetic and conjugated nanospheres showed almost the same amount (780–782 ICG dye per nanoparticle) of fluorophore encapsulation, viz., ICG dye, in their deep core, as shown in Figure 3A–H. Over 30 days of storage, biomimetic nanospheres (740 MESF unit) showed better ultrabrightness than nanospheres (690 MESF unit), which may be due to the strong protection of encapsulated ICG from its sensitive probes/molecules. Moreover, both biomimetic and conjugated nanospheres showed better photostability (740 and 728 MESF unit) than free ICG dye molecules (98 MESF unit), as shown in Figure 3B,H. It should be noted that quantum dots are very popular for imaging applications, but their high brightness can only be achieved after assembling several quantum dots. Quantum dots quench their fluorescence when the distance between them is less than 10 nm,^63^ but this was not the case for our designed nanospheres due to the unique self-assembly of cellulose acetate with the folic acid-conjugated guest polymer PF127, where ICG dye molecules were encapsulated in the core of particles with a diameter of 180–240 nm with a perfect distance (more than 10 nm). On the other hand, these observations were validated through microscopic imaging, where nanoparticles (biomimetic and conjugated nanospheres) exhibited controlled particle morphology (spherical) and size (180–240 nm) on the 1st and 30th day of preparation, as shown in Figure 3C,D,I,J. From single-photon microscopic imaging, each bright dot represents a few aggregated large nanoparticles (ICG dye-encapsulated biomimetic and conjugated nanospheres). Remarkably, it should be noted that the obtained nanoparticles (ICG dye-encapsulated biomimetic and conjugated nanospheres) maintained their brightness even after storage for 30 days (Figure 3E,F,K,L).

**Figure 3:**
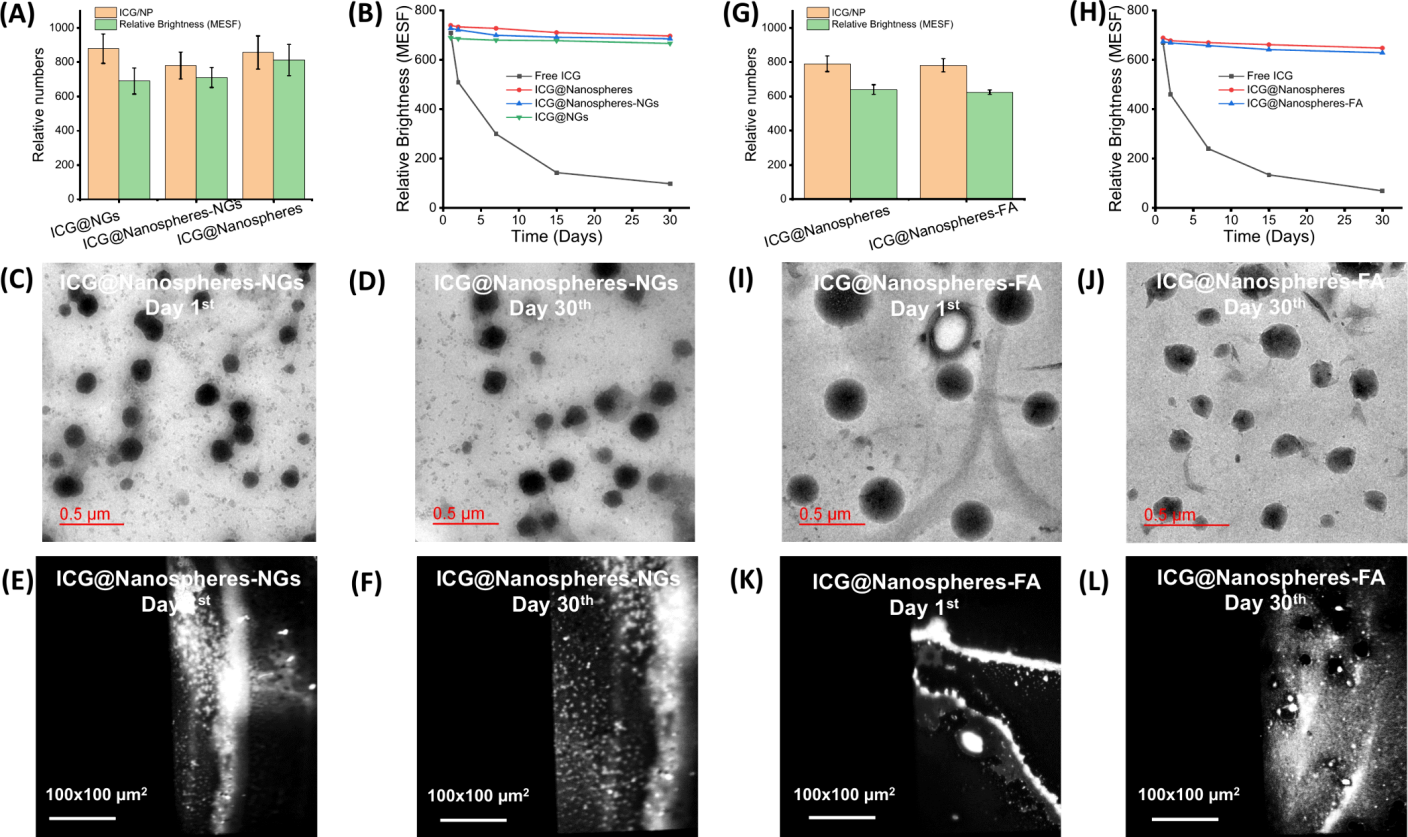
(A, B, G, and H) Relative brightness of biomimetic and folic acid-conjugated ultrabright nanoparticles in terms of number of dye molecules per nanoparticles, (C, D, I, and J) TEM images of biomimetic and folic acid-conjugated ultrabright nanoparticles at 1st day and 30th days of preparation, and (E, F, K, and L) single-photon microscopic images of biomimetic and folic acid-conjugated ultrabright nanoparticles at 1st day and 30th days of preparation showing brightness of nanoparticles. Each bright dots represent a few (3–4) aggregated nanoparticles.

Overall, the calculated values suggest that a single nanoparticle accommodates a few hundred dye molecules exhibiting maintained brightness for a prolonged period (tested up to 30 days), demonstrating the photostability of emissive nanoparticles as stable imaging agents, whereas free dye molecules demonstrated more than 30% degradation in emission.

### Biocompatibility, Targeted Cancer Cell Imaging, and Chemotherapy

Prior to targeted cancer cell imaging and chemotherapeutic applications, the prepared biomimetic and folic acid-conjugated nanoparticles were tested for biocompatibility and hemocompatibility at various concentrations (5–200 μg/mL), as shown in Figure 4A,B. The MTT assay on healthy cells, viz., L929 fibroblasts, indicated more than 92% cell viability even at the highest concentration (200 μg/mL) of nanoparticles and related parent constituents, viz., nanospheres, nanospheres-FA, nanospheres-NGs, ICG@nanospheres, ICG@NGs, DOX@nanospheres-FA, DOX@nanospheres-NGs, etc. Free DOX exhibited only 15% cell viability even at the lowest concentration (5 μg/mL), healthy cells could not survive at moderate (50 μg/mL) and maximum (200 μg/mL) concentrations of free DOX due to the highly toxic nature of the anticancer drug (Figure 4A). On the other hand, at all concentrations (5–200 μg/mL), biomimetic nanoparticles such as NGs, ICG@NGs, and DOX@NGs demonstrated better hemocompatibility (more than 84%) due to natural surface biomarkers, whereas other nanoparticles such as ICG@nanospheres, ICG@ nanospheres-FA, and DOX@ nanospheres-FA showed low hemocompatibility (60% at low concentration, viz., 5 μg/mL to 11% only at high concentrations, viz., 200 μg/mL) due to strong interaction between negatively charged functional groups of nanoparticles and the choline group of red blood cells (Figure 4B). However, the mechanism of hemocompatibility of biomimetic nanoparticles has not been well studied. Accounting for biocompatibility examinations, the engineered nanoparticles were found to be suitable for further cancer cell diagnosis and targeted cancer therapeutics studies. Doxorubicin (DOX), an anticancer drug, was entrapped within the engineered nanoparticles for targeted cancer cell imaging and chemotherapeutic studies, as shown in Figure 4C,D. Cell-membrane-based biomimetic^64–66^ nano ghost vesicles demonstrated better loading efficiency (48%) than engineered nanospheres (34%), which may be due to the large cavity in the ghost nanovesicles. DOX loading in nanoparticles was characterized through spectroscopic measurements, showing a broad peak of absorption around 510 nm and emission around 582 nm, as shown in the Supporting Information, Figure S3A,B. The DOX-loaded biomimetic and folic acid-conjugated nanospheres were incubated with breast cancer cells, viz., MCF-7. After 6 h of incubation, DOX-entrapped biomimetic nanospheres demonstrated a homogeneous distribution of red fluorescence in the cancer cell interior, indicating the rapid uptake of nanoparticles due to natural surface biomarkers, which play a vital role in cell targeting (Figure 4C). However, the mechanism of biomimetic nanoparticle targeting is not yet well-known. It should be noted that free DOX easily diffuses into the cells (cancerous and noncancerous) even at low concentration, which further enter into the nucleus for inhibiting DNA and RNA polymerase for ultimately cell death. On the other hand, DOX-loaded folic acid-conjugated nanospheres demonstrated significant red fluorescence from the cancer cell interior, but the distribution was heterogeneous even after incubation for 6 h, which indicated the slow release of fluorescent DOX from the dense polymeric network of conjugated nanospheres (Figure 4C). The targeting mechanism of folate-modified nanospheres is based on the interaction between folate groups and overexpressed folate receptors on the cancer cell membrane. Similar fluorescence-based observations were also observed in the case of ICG (red-emissive dye)-loaded nanoparticles (biomimetic and folic acid-conjugated), as discussed in the text below.

**Figure 4:**
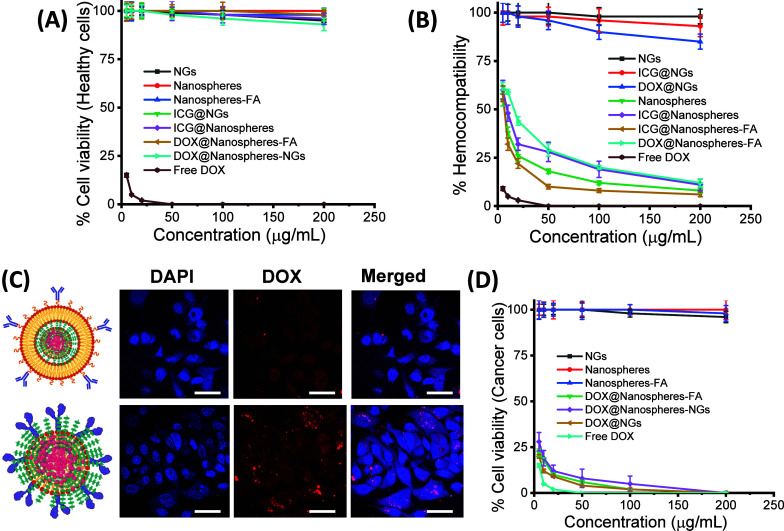
(A, B) % Cell viability and hemocompatibility of biomimetic and folic acid-conjugated ultrabright nanoparticles and their related nanoparticulate formations (NGs, nanospheres, nanospheres-NGs, nanospheres-FA, etc.) with and without drug loading, (C) targeted breast cancer cell imaging of DOX-encapsulated biomimetic and folic acid-conjugated ultrabright nanoparticles after 6 h of incubation, and (D) targeted chemotherapeutics response of biomimetic and folic acid-conjugated ultrabright nanoparticles and their related nanoparticulate formations (NGs, nanospheres, nanospheres-NGs, nanospheres-FA, etc.) where without drug loading nanoparticles are considered as control groups.

To test potential anticancer activity, the DOX-loaded nanoparticles (biomimetic and folic acid-conjugated) were incubated with breast cancer cells (MCF-7) at various concentrations (5–200 μg/mL). The used MTT assay demonstrated that DOX-entrapped biomimetic cell-membrane nano ghost (NGs) resulted in more than 96% cell death at a low concentration (50 μg/mL), whereas DOX-entrapped folic acid-conjugated nanospheres exhibited approximately 94% cell death at a 50 μg/mL concentration (Figure 4D). Because of the inherent surface biomarkers, DOX-loaded biomimetic nanospheres (DOX@nanospheres-NGs) exhibited promising cell death (approximately 83%) even at a lower concentration (5 μg/mL), which was higher than that of folic acid-conjugated DOX@nanospheres at a 5 μg/mL concentration. These significant cell deaths indicated the high cellular uptake of drug-loaded nanoparticles, which released loaded anticancer cargo DOX within the cancer cell interior. It should be noted that the strong cellular binding of nanoparticles was due to natural surface biomarkers and folic acid-targeting ligands, as corroborated by another set of cell imaging studies in which ICG dye was used as a fluorescent agent. In brief, red-emissive ICG dye-encapsulated biomimetic and folic acid-conjugated nanospheres were incubated with MCF-7 cancer cells for 6 and 12 h, as shown in Figure 5. Biomimetic fluorescence nanospheres (ICG@ nanospheres-NGs) showed augmented homogeneous red fluorescence distribution in the cancer cell interior within 6 h of incubation, which was improved with respect to the incubation time (12 h), indicating the rapid uptake and long-term imaging effect of nanoparticles due to natural surface biomarkers of coated biomimetic cell membranes on the polymeric nanospheres, which might play a vital role in cell targeting. Folic acid-conjugated fluorescent nanoparticles (ICG@nanospheres-FA) showed red emission with a heterogeneous distribution from the cancer cell interior after 6 h of incubation, which was further examined after 12 h of incubation. However, insignificant improvements in fluorescence distribution from cancer cells were observed even after long incubation periods, indicating low cellular uptake compared with biomimetic nanoparticles.

**Figure 5:**
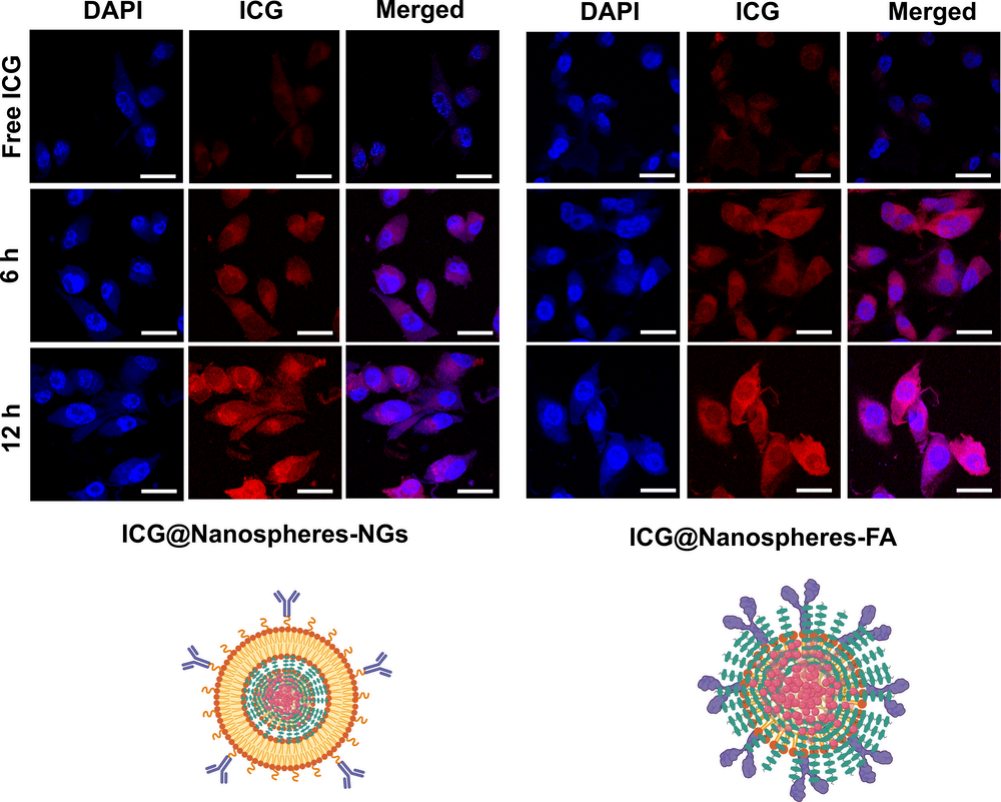
Targeted breast cancer cell imaging of ICG dye-encapsulated biomimetic and folic acid-conjugated ultrabright nanoparticles after 6 and 12 h of incubation.

### Stimuli-Responsive Cargo Delivery and Image-Guided Chemotherapy

Cargo-loaded (48% DOX drug and 52% ICG dye) biomimetic and folic acid-conjugated nanospheres were tested for release kinetics under various cellular conditions (pH 4–6.8) and compared with the physiological environment (pH 7.4), as shown in Figure 6. Biomimetic nanoparticles showed significant drug release (80 ± 5%) in a highly acidic environment (pH 4) within a short period of kinetics (only 8 h), whereas folic acid-conjugated nanoparticles showed 78 ± 2% drug release at the same incubation time (Figure 6A,B). Furthermore, biomimetic nanoparticles improved the release of the therapeutic drug to 95% in just 12 h of incubation, whereas conjugated nanoparticles demonstrated approximately 88 ± 2% release. It should be noted that nearly 100% of drug molecules were released at lower pH (4) in 24 h of kinetics time for both the nanoparticles, indicating the degradation or disassembly of engineered polymeric nanospheres due to protonation of functional groups within polymeric chains at acidic conditions. From the release kinetic patterns, we understood that folic acid-conjugated PF127 strengthened the polymeric network shield within cellulose acetate macromolecules (conjugated nanospheres), where drug molecules were strongly entrapped, which slowed the release of entrapped cargo molecules. To the best of our knowledge, the strong and dense polymeric network hampered the release of the loaded chemotherapeutic cargo in a cancer-mimicking environment, which was almost similar to the release performance under neutral conditions (extracellular pH 7.4). The release kinetics of the engineered nanoparticles was also evaluated using ICG dye as cargo molecules, where these dye molecules were trapped in the deep core of each nanoparticle. Under strongly acidic conditions (pH 4), that is, deep intracellular pH of cancer cells, biomimetic nanospheres showed more than 95% ICG release in just 6 h of incubation due to protonation of self-assembled CA and PF127 and coated cell-membrane ghost nanovesicles (Figure 6C). Moreover, these particles exhibited almost 100% dye release within 12 h of incubation at pH 4, indicating that these biomimetic nanoparticles are suitable for cargo delivery/chemotherapeutic applications. It should be noted that less than 20% of the dye was released at pH 6, which was significantly higher at pH 5.4 (88 ± 4%) and pH 4 (96 ± 2%) after 24 h of incubation. In contrast, ICG-entrapped folic acid-conjugated nanoparticles (ICG@ nanospheres-FA) exhibited approximately 64 ± 3% dye release at pH 5.4 after 24 h of incubation, which was further improved to 79 ± 4% at pH 4 in the same incubation time (Figure 6D). Furthermore, these particles demonstrated more than 90 ± 3% dye release in just 12 h of incubation in acidic conditions, i.e., cancer-mimicked environment, and less than 20 ± 2% release was noted at pH 6 and pH 7.4, indicating the stimuli-responsive nature of the engineered nanoparticles. Based on stimuli-responsive cargo delivery (both DOX and ICG), the engineered nanoparticles (biomimetic as well as conjugated nanospheres) were examined for targeted chemotherapeutic applications. Briefly, DOX-loaded nanoparticles (both biomimetic and folic acid-conjugated CA-dots) were incubated with breast cancer cells, viz., MCF-7, for 6 h, where better cellular uptake was observed. As discussed above, biomimetic nanoparticles demonstrated better uptake with a homogeneous distribution of therapeutic cargo molecules within cancer cells compared to folic acid-conjugated CA-dots. Similarly, in targeted chemotherapeutics, DOX-loaded biomimetic nanospheres exhibited a rapid release of chemotherapeutic drugs that killed cancer cells, representing their altered morphology, as shown in Figure 7. In contrast, cancer cell morphology was maintained even after treatment with DOX-loaded folic acid-conjugated nanospheres, indicating their poor death due to the slow release of loaded chemotherapeutic drugs in the cancer cell interior. These observations were corroborated by stimuli-responsive drug release, targeted cancer cell imaging, and *in vitro* therapeutic testing. Overall, the quick cell uptake and strong targeting of imaging and therapeutic particles have become an urgent need for early stage diagnosis and treatment of cancer. Moreover evaluating the effectiveness, i.e., long-term stay of the tested nanoparticle-based imaging and therapeutics system in cancer-mimicked environment, the biomimetic (cell-membrane-coated nanoparticles) nature of nanoparticles helps them to evade immune cell reorganization and uptake upon administration into the blood circulation. However, the *in vivo* examinations are beyond the scope of this work.

**Figure 6:**
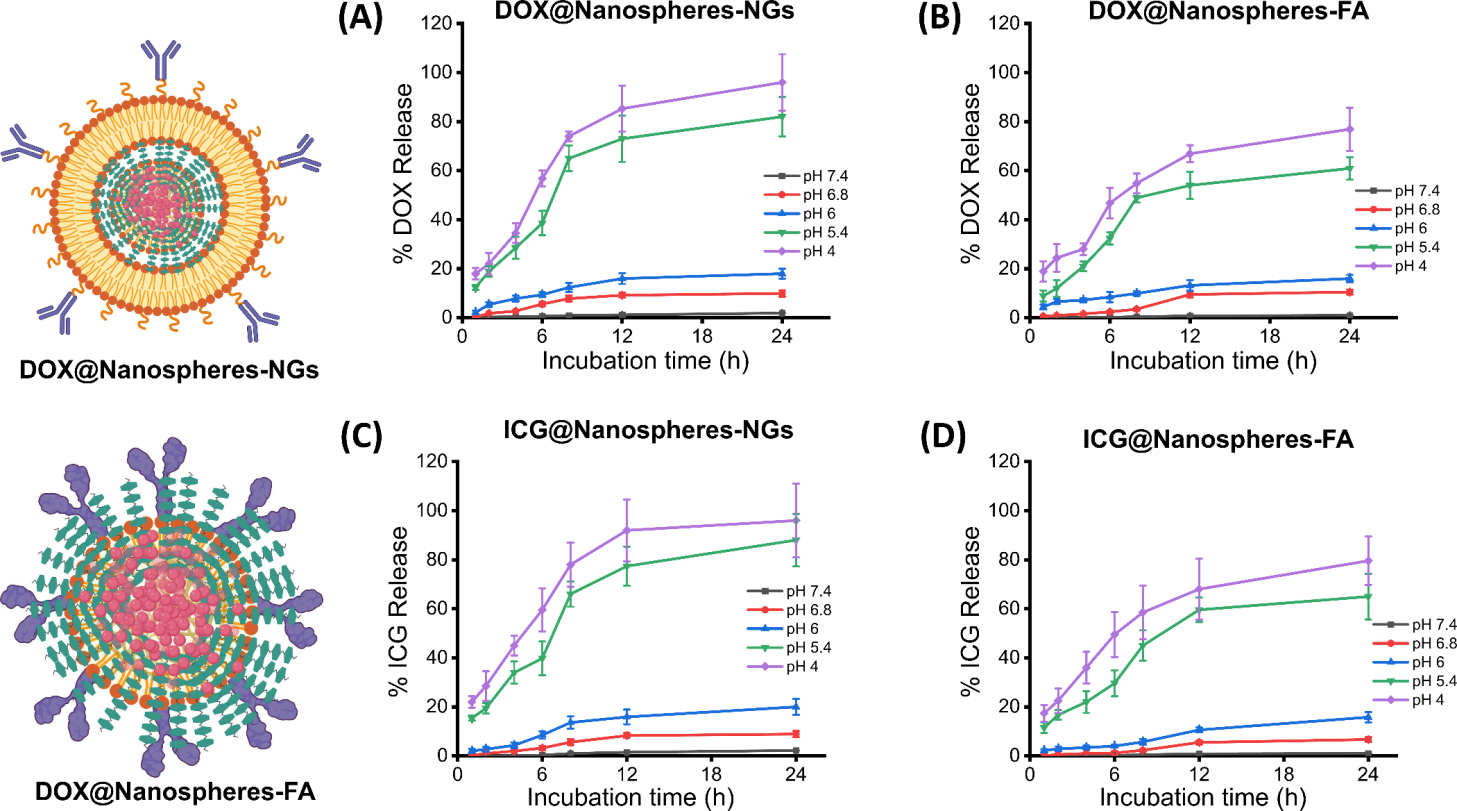
Time-dependent (A, B) DOX drug and (C, D) ICG dye release kinetics of DOX-encapsulated biomimetic and folic acid-conjugated ultrabright nanoparticles at various conditions of cancer cell interior (pH 4–6.8) comparing with an extracellular condition (pH 7.4).

**Figure 7:**
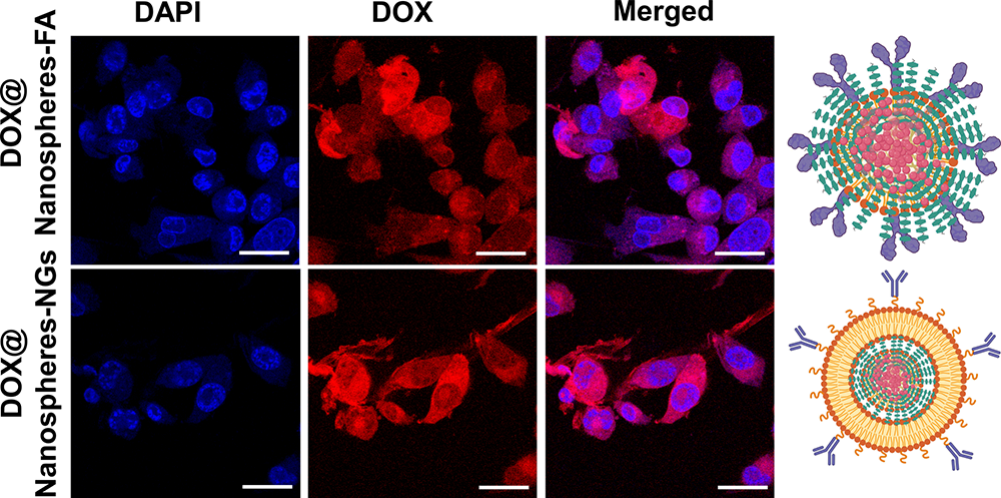
Targeted chemotherapeutics effect of DOX-encapsulated biomimetic and folic acid-conjugated ultrabright nanoparticles after 6 h treatment of cancer cell. The changes in cell morphologies clearly indicate the image-guided therapeutics response of treated chemotherapeutics nanoparticles.

## Conclusions

It should be noted that early stage diagnosis and treatment of cancer are the primary needs in human healthcare. Therefore, an ideal imaging and therapeutic system should perform its response quickly in the cancer-mimicked environment. The present work describes the cost-effective engineering of safe and reliable ultrabright nanoparticles (biomimetic and conjugated nanospheres) for early stage cancer diagnosis and therapeutics. The ultrabright and biomimetic nature of nanoparticle systems helps to identify the cancer cells locally by escaping from immune cell recognition. Such parameters boost the preclinical and clinical translational of nanosized imaging and therapeutics medicines. In this project, biomimetic and conjugated spherical polymeric nanoparticles (180–240 nm in size) have been achieved through one-step encapsulation of fluorescent dyes and drugs in their hydrophobic core. The specific arrangement and strong entrapment of fluorescent organic dyes in the polymeric deep core are key factors in achieving ultrabrightness of engineered nanoparticles. Our findings suggest that folic acid-conjugated PF127 moieties strengthen the polymeric network of the prepared nanospheres with strong entrapment of loaded cargo (fluorescent dye and drug) molecules in their hydrophobic core. Separately prepared biomimetic nanoghosts from cancer cell membranes have been used to cover polymeric nanospheres. These surface-coated/conjugated ultrabright nanoparticles (780–980 MESF) have been validated through spectroscopic and microscopic characterization. Furthermore, various essential parameters such as colloidal stability, photostability, biocompatibility, scalability, targeting ability, etc. have been examined. Most importantly, the engineered ultrabright nanoparticles, i.e., conjugated and biomimetic, exhibit targeted cancer cell imaging and chemotherapeutics at the minimum concentration, indicating the scope of *in vivo* studies with a low-dose requirement. Hence, such advantages open a direction for further preclinical and clinical validations of the engineered ultrabright nanoparticles. The quick stimuli response of these cargo-encapsulated nanoparticles in cancer-mimicking environments ensures their effectiveness for localized chemotherapeutics application. Overall, based on targeted cell imaging and therapeutics outcomes, we have understood that biomimetic nanoparticles demonstrate better accumulation and release of cargo molecules in the cancer cell interior than the folic acid-conjugated nanoparticles, indicating the importance of natural surface biomarkers over folic acid as a targeting ligand. In addition, the promising cancer cell death represents the targeted chemotherapeutic capability of biomimetic nanoparticles, which may evade the uptake of immune cells for site-selective targeting.

## Methods

### Ultrabright Conjugated Polymeric Nanodots

Ultrabright polymeric nanoparticle (ICG dye-encapsulated cellulose acetate (CA) network and polymeric guest PF127) molecules were prepared using nanoprecipitation at room temperature and pressure. Prior to conjugation, the folic acid-targeting ligand was conjugated with the guest polymeric chain, PF127. For this coupling conjugation, dried folic acid (FA, 0.5 mM) was mixed in DMSO and activated with 0.2 mM carboxydiimidazole (CDI) followed by an overnight reaction at room temperature in the dark. Next, predried Pluronic F127 (PF127, 0.1 mM) was added to the solution, and the reaction proceeded overnight. Folic acid-conjugated PF127 polymeric chains (PF127-FA) were purified by overnight dialysis. These conjugated polymeric chains were used to produce ultrabright polymeric nanoparticles. Briefly, the organic phase (0.01 mM ICG dye and 5 mg/mL cellulose acetate in 5 mL of acetone) and aqueous phase (2 mg/mL PF127-FA in a total of 15 mL of Milli-Q water) were mixed at high stirring speeds to achieve nanoprecipitation. To prepare the organic phase, cellulose acetate CA (30 K molecular weight) in an organic solvent was premixed with high stirring (900 rpm) and sonicated for 40 s at room temperature for 6 h. After 6 h of mixing, 0.01 mM ICG dye was added to the organic CA solution and the mixture was mixed for an additional 2 h. An aqueous phase of PF127-FA was prepared in Milli-Q water at pH 7.4 using overnight mixing at room temperature (RT). The prepared organic phase (the CA and ICG mixture) was quickly introduced into the aqueous phase (PF127-FA) under high stirring (700–900 rpm) at room temperature. After rapid mixing of both phases, the obtained mixture was stirred overnight at room temperature in the dark and then kept in a vacuum oven at 50 °C to evaporate the organic solvent, acetone. The remaining solvent was removed by dialyzing the nanoprecipitated solution (where nanoparticles were formed) for 2 days using 12–14 kDa dialysis membranes against Milli-Q water. During dialysis, the dialysate was changed every 6 h (for daytime) and 12 h (for night time), and the obtained particles were filtered using a syringe filter before characterization. Organic solvent-mixed DOX was used to prepare nanoparticles for chemotherapeutic applications, and the remaining procedure was the same as that mentioned above.

#### Ultrabright Biomimetic Nanodots

Colon cancer cells (Caco-2, human epithelial cells) were used to prepare biomimetic ghost nanoparticles, followed by a biochemical hypotonic treatment through sonication. Caco-2 cells (2 × 10^5^) were centrifuged and washed with ice-cold tris-magnesium buffer (containing 10 mM Tris and 1 mM MgCl_2_) using centrifugation (10,000 rpm), and the collected cells were resuspended in 15 mL of 0.25 M sucrose containing tris-magnesium buffer. The suspended cells were homogenized and sonicated for 15 min in an ice bath. These cells were homogenized and subjected to freeze–thawing for 6 h. The fractured cells were diluted with a mixture of PBS and sucrose containing tris-magnesium buffer (to a total volume of 20 mL) and centrifuged five times to separate the supernatant and pellet. The obtained pellet (cell ghosts) was centrifuged, washed with 0.25 M sucrose containing tris-magnesium buffer, further sonicated (15 cycles) with 20% intensity and 5 s on/off the pulse, and collected via centrifugation using sucrose containing tris-magnesium buffer. The aqueous suspension of the collected pellet (containing the ghosts) was sonicated for 10 min at a 20% amplitude and centrifuged for 20 min. Finally, the supernatant (membrane ghost nanovesicles) was collected and mixed with the ICG dye and DOX drug-encapsulated polymeric nanospheres. After overnight mixing, the prepared mixture was filtered under mild pressure and centrifuged for 1 h to collect biomimetic ultrabright nanoparticles. The obtained nanoparticles were resuspended in PBS for further use.

#### Ultrabrightness Measurements

An Agilent fluorimeter and UV–vis Cary 60 Spectro photometer were used to measure the brightness, fluorescence, and photostability. The molecules of equivalent soluble fluorophores were used to measure the brightness of the designed nanoparticles. The relative brightness was measured by comparing the brightness of fluorescent-dye-encapsulated nanoparticles and fluorescent dye molecules with a known brightness within a close spectrum. Mainly, DLS measurements were applied to check the weight of nanoparticles in a known volume of nanoparticle dispersion and a related concentration. The following equations were used to calculate the brightness of the single nanoparticle:

$$\mathrm{weight}\;\mathrm{of}\;one\;\mathrm{NP}\;(\mathrm{mg})=\rho \times \frac{4}{3}\pi r^{3}$$ 1

$$\mathrm{number}\;\mathrm{of}\;\mathrm{NPs}\;/\mathrm{mL}=\frac{C}{\left(\rho \times \frac{4}{3}\pi r^{3}\right)}$$ 2

$$\mathrm{number}\;\mathrm{of}\;\mathrm{dye}\;\mathrm{molecules}/\mathrm{mL}=\frac{A}{\varepsilon }\times 6.023\times 10^{23}$$ 3

$$\mathrm{relative}\;\mathrm{brightness}=\frac{\left(\mathrm{fluorescense}\;\mathrm{of}\;\mathrm{NPs}/\mathrm{conc.}\;\mathrm{of}\;\mathrm{NPs}\right)}{\left(\mathrm{fluorescense}\;\mathrm{of}\;\mathrm{dye}/\mathrm{conc.}\;\mathrm{of}\;\mathrm{dye}\right)}$$ 4

### Biocompatibility, Targeted Cancer Cell Imaging

Biocompatibility of the prepared nanoparticles was ensured via *in vitro* cytotoxicity and hemocompatibility at various concentrations (5–200 μg/mL), followed by MTT and optical spectroscopic measurements. Various formulations of designed ultrabright nanoparticles (NGs, nanospheres, nanospheres-FA, ICG@NGs, ICG@ nanospheres, DOX@nanospheres-FA, and DOX@nanospheres-NGs) were used for biocompatibility studies and compared with their control, that is, free DOX and ICG molecules. In the *in vitro* MTT assay, L929 cells (5 × 10^5^ cells per treatment) were cultured as per the standard protocol25, treated with biomimetic and conjugated nanoparticles using 100 μL of each nanoparticle at various concentrations (5–200 μg/mL), and then washed with PBS to remove excess particles. Next, 20 μL of MTT dye was added to check the % cell viability in the above treatments, and the formed formazan crystals were dissolved in 200 μL of DMSO. The optical absorbance was recorded by using a microplate reader (Tecan Infinite 200 PRO). Untreated cells were used as a control to calculate the % cell viability using the MTT assay. The hemocompatibility of the nanoparticles was measured in healthy red blood cells. Healthy red blood cells (1 mL) were diluted with PBS (total volume of 15 mL) and washed thoroughly with PBS, followed by centrifugation. The collected RBCs were treated with various concentrations of biomimetic and conjugated nanoparticles (5–200 μg/mL) for 12 h (200 μL of RBCs and 800 μL of nanoparticles at each concentration). After 12 h of treatment, the mixtures were centrifuged at 5000 rpm for 20 min, and the absorption of released hemoglobin at 540 nm was measured by spectroscopic analysis. The percentage of the hemolysis was calculated using the following equation.^25^

$$\%\;\mathrm{hemolysis}=\frac{\left(A_{\mathrm{sample}}-A_{\mathrm{negative\_control}}\right)}{\left(A_{\mathrm{positive\_control}}-A_{\mathrm{negative\_control}}\right)}\times 100\%$$

where *A*_sample_, *A*_positive_control_, and *A*_negative_control_ are the absorbances of the sample, positive control and negative control, respectively.

To examine the targeting of cancer cells, human breast epithelial cancer cells (MCF-7, 5 × 10^5^ cells/well) were cultured in DMEM supplemented with 10% fetal bovine serum and penicillin/streptomycin for 24 h of incubation in 5% CO_2_ at 37 °C. Next, 100 μL of ICG dye-encapsulated biomimetic and folic acid-conjugated nanoparticles were incubated with the breast cancer cells for 6 and 12 h. After complete incubation, unencapsulated nanoparticles were removed by washing the treated cancer cells with PBS. Then, the cells were fixed with 4% paraformaldehyde and stained with 4,6-diamidino-2-phenylindole (DAPI for cell nuclei). The stained cells were mounted on a glass slide using 70% glycerol and a coverslip and scanned using a fluorescence microscope to track the location of red fluorescence, which was due to the red-emissive ICG dye and blue nuclei due to DAPI staining.

### Stimuli-Responsive Cargo Delivery and Targeted Chemotherapeutics

A 10 mL suspension of polymeric nanoparticles with encapsulated doxorubicin anticancer drug and ICG dye was placed in a dialysis bag, which was immersed in 100 mL of PBS with various pH values (4–7.4). At different time points (1,2, 4, 6, 8, 12, and 24 h), 5 mL of the solution was collected from the dialysate and replaced with the same volume of fresh PBS solution, that is, the respective pH, to maintain a constant volume. The release kinetics were evaluated for 24 h at a physiological 37 °C temperature in the dark. UV–vis–NIR spectroscopic measurements were performed to evaluate the amount of the anticancer DOX drug (510–512 nm) and ICG dye (779–785 nm) in the dialysate medium. For targeted chemotherapeutic response, breast cancer cells (MCF-7, 5 × 10^5^ cells per treatment) were treated with various concentrations of doxorubicin anticancer drug-encapsulated biomimetic and folic acid-conjugated nanoparticles, with DOX-free nanoparticles as the control. After 24 h of culture, breast cancer cells were treated with drug-loaded nanoparticles (100 μL of 5–200 μg/mL particle dispersion) for 6 h and then washed with PBS to remove untaken particles. The MTT assay was used to evaluate the % cell viability by dissolving formazan crystals in DMSO (200 μL). The optical absorbance was recorded using a microplate reader (Tecan Infinite 200 PRO). Microscopic images were captured to evaluate the changes in cell morphology.

## Supplementary Material



## Data Availability

The data will be made available upon request. Some data cannot be shared at this time owing to technical or time limitations, and the data form part of an ongoing study.

## Notes

TEM images, optical spectra, FTIR spectra, and relative brightness measurements; all experiments were performed in triplicate; all data were analyzed and plotted using OriginPro 8, GraphPad, and Sigma plot software (version 10.0); and significant differences between groups were calculated using *t* test (PDF)
