# Supplementary material for: Long-Term Cell-Membrane-Coated Ultrabright Nanospheres for Targeted Cancer Cell Imaging and Hydrophobic Drug Delivery
Source: Chem Mater. 2025 Jan 30;37(3):845–56. doi: 10.1021/acs.chemmater.4c01819 (PMC12818752; doi:10.1021/acs.chemmater.4c01819)
Supplement: Supplementary file 1 [file cm4c01819_si_001.pdf]

Supporting information

Long-term cell-membrane coated ultrabright nanospheres for targeted cancer cell imaging and hydrophobic drug delivery

Rajendra Prasad[a, b]*, Berney Peng[b], Narendra Gupta[c], Avtar Singh Meena[d], Geetha Satya Sainaga Jyothi Vaskuri[e], Anuj Chandak[c], Igor Sokolov[b]*, João Cond[f,g] *

[a]School of Biochemical Engineering, Indian Institute of Technology (BHU) Varanasi, Uttar Pradesh 221005, India

[b]Departments of Mechanical Engineering and Biomedical Engineering, Tufts University, Medford, Massachusetts, 02155, USA

[c]Vasco Healthcare Pvt. Ltd., Gopal Pura Bai Pass Jaipur, Rajasthan, 303006, India

[d]Department of Biotechnology, All India Institute of Medical Sciences, Ansari Nagar, New Delhi-110029, India

[e]Pharmaceutical Sciences Department, College of Pharmacy, University of Tennessee Health Science Centre, Memphis, Tennessee, 38163, USA

[f]NOVA Medical School|Faculdade de Ciências Médicas, NMS|FCM, Universidade NOVA de Lisboa, Lisbon 1169-056, Portugal

[g]ToxOmics, NOVA Medical School|Faculdade de Ciências Médicas, NMS|FCM, Universidade NOVA de Lisboa, Lisbon, 1169-056, Portugal

*** Corresponding authors: rajendra.bce@iitbhu.ac.in ; igor.sokolov@tuft.edu; joao.conde@nms.unl.pt**

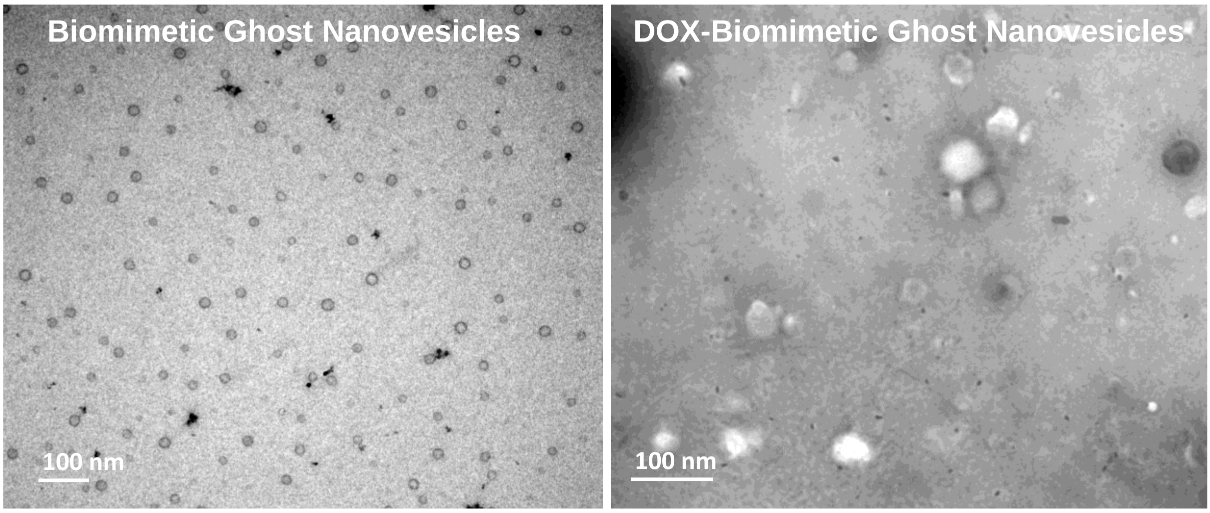

Figure S1. TEM images of cancer cell membrane derived biomimetic ghost nanovesicles with and without DOX drug entrapment.

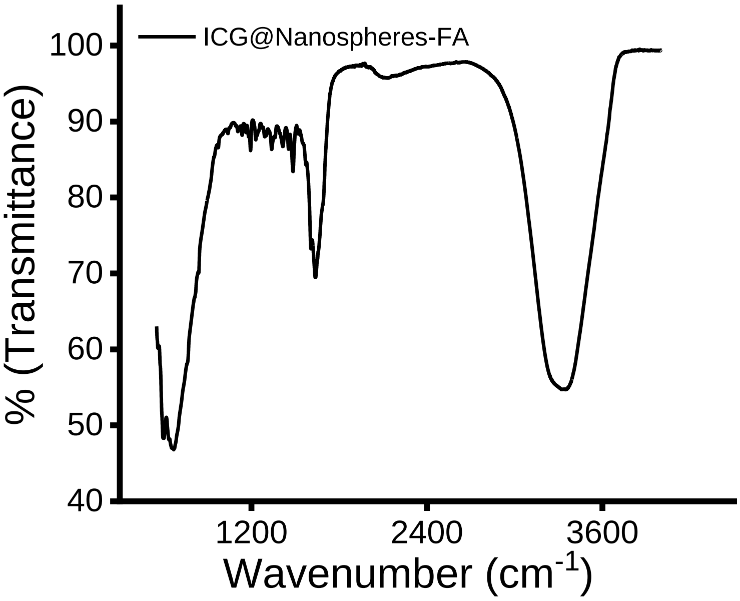

Figure S2. FTIR spectrum of folic acid conjugated ultrabright nanospheres.

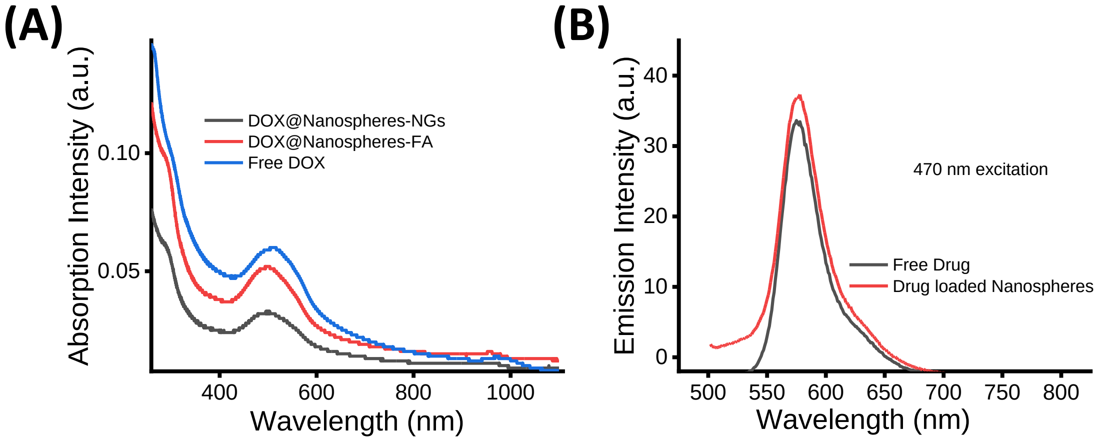

Figure S3. (A) UV-Vis absorption spectra of drug (DOX) loaded folic acid conjugated nanospheres, biomimetic nanospheres and free DOX and (B) emission spectra of free drug and drug loaded nanospheres.
